# Exposure to Conflict-Related News and Psychological Distress Among Nursing Students: The Mediating Role of Sleep Difficulties and Study Disruption

**DOI:** 10.3390/healthcare14121609

**Published:** 2026-06-08

**Authors:** Majed M. Aljabri, Bandar S. Alharbi, Endale Alemayehu Ali

**Affiliations:** 1 Community and Psychiatric Mental Nursing Health Department, College of Nursing, King Saud University, Riyadh 12375, Saudi Arabia; banalharbi@ksu.edu.sa; 2 Department of Public Health and Primary Care, KU Leuven, Kapucijnenvoer 33, 3000 Leuven, Belgium

**Keywords:** conflict exposure, mental health, nursing students, depression, anxiety, social media, psychological distress

## Abstract

**Background:** Armed conflict and geopolitical instability increasingly affect mental health beyond directly exposed populations through continuous media exposure and digital information dissemination. Nursing students may be particularly vulnerable because of high academic demands, emotional sensitivity to human suffering, and intensive engagement with social media and online news platforms. This study examined the association between conflict related news exposure and depression, anxiety, and stress among nursing students in Saudi Arabia during the February 2026 regional military escalation involving Iran, and explored the role of perceived safety concern and the potential indirect associations involving sleep difficulty and study disruption. **Methods:** A multi-center cross sectional study was conducted among nursing students from different public universities across five regions of Saudi Arabia. Psychological distress was assessed using the Depression Anxiety Stress Scale. A composite conflict exposure index was developed from conflict news following frequency and exposure intensity measures. We used Gamma generalized linear models, interaction analyses, and structural equation modeling to evaluate associations, moderation by information source, and mediation pathways. Results were reported as arithmetic mean ratios (AMRs) with 95% confidence intervals, representing the relative change in mean psychological distress scores associated with each predictor. Models were adjusted for sociodemographic, academic, and living-related factors. **Results:** A total of 247 nursing students were included. Moderate to severe depression, anxiety, and stress were reported by 50.2%, 59.9%, and 32.4% of participants, respectively. Our findings showed that higher conflict exposure was associated with increased stress levels (AMR = 1.17, 95% CI: 1.02–1.34), while associations with depression (AMR = 1.14, 95% CI: 0.99–1.30) and anxiety (AMR = 1.13, 95% CI: 0.99–1.28) were weaker. Associations between conflict-related exposure and depression, anxiety, and stress were substantially attenuated after accounting for perceived safety concern, which remained strongly associated with all psychological distress outcomes (AMR = 1.32, 95% CI: 1.19–1.47), anxiety (AMR = 1.31, 95% CI: 1.18–1.44), and stress (AMR = 1.36, 95% CI: 1.24–1.51). Compared with television news users, students relying on online news demonstrated substantially higher depression (AMR = 1.92, 95% CI: 1.32–2.78), anxiety (AMR = 1.84, 95% CI: 1.29–2.64), and stress scores (AMR = 1.88, 95% CI: 1.29–2.74). Structural equation modeling identified significant indirect associations involving sleep difficulty and study disruption, whereas direct associations between exposure and psychological distress were comparatively weak. **Conclusions:** Conflict-related media exposure was associated with poorer mental health among nursing students, with perceived insecurity, sleep difficulties, and study disruption showing strong associations with psychological distress and patterns consistent with indirect relationships. Universities and nursing education programs should consider implementing mental health support, media literacy interventions, sleep health promotion, and psychosocial support strategies during periods of regional geopolitical instability.

## 1. Introduction

Mental health disorders represent a major and growing global public health challenge. According to the World Health Organization, nearly 1.1 billion people worldwide were living with a mental disorder in 2021, with anxiety and depressive disorders accounting for a substantial proportion of the global burden of disability [1]. Young adults are particularly vulnerable to the onset and progression of mental health conditions because this developmental period is characterized by substantial academic, emotional, social, and occupational transitions [2,3]. University students frequently experience elevated levels of psychological distress related to academic workload, financial pressure, social adjustment, and uncertainty regarding future careers. Nursing students may be especially vulnerable because of the additional demands of clinical education, exposure to patient suffering, emotional labor, and prolonged academic intensity. Recent studies indicate that university students experience considerably higher levels of psychological distress than the general population, with anxiety and depressive symptoms being especially common among students in medical and nursing programs [4,5].

In recent years, geopolitical instability and armed conflicts across the Middle East have emerged as additional psychological stressors affecting populations throughout the region. Armed conflict is increasingly recognized not only as a humanitarian and political crisis but also as a major determinant of population mental health [6]. Continuous exposure to casualty reports, political uncertainty, and emotionally charged narratives may generate chronic fear, emotional exhaustion, hypervigilance, and generalized psychological distress even among individuals geographically distant from active conflict zones [7].

The recent escalation of military tensions and armed conflict involving Iran generated extensive media coverage throughout the Middle East and globally. In Saudi Arabia and neighboring Gulf countries, news related to missile attacks, military escalation, regional instability, and security concerns became highly visible within daily information environments. Given Saudi Arabia’s geopolitical proximity and strategic position within the region, the conflict carried substantial psychological salience among the general population. University students were repeatedly exposed to conflict-related content through television broadcasts, online news portals, and social media platforms including X, TikTok, Instagram, Telegram, and Snapchat. Unlike conventional broadcast media, social media platforms frequently amplify emotionally intense content through algorithm-driven repetition, graphic visual material, sensational framing, and continuous public commentary [8].

The psychological effects of media-mediated exposure to conflict have been documented across multiple international crises. Recent evidence among healthcare students and nursing students exposed to conflict environments further suggests that healthcare trainees may be particularly vulnerable to conflict-related psychological burden because of elevated empathic engagement with suffering populations [7]. The psychological wellbeing of nursing students is shaped by a complex interplay of both protective and risk factors. Recent evidence suggests that factors such as self-efficacy, cognitive flexibility, and adaptive coping may enhance resilience, whereas exposure to stressful information environments, sleep disturbances, and academic challenges may increase vulnerability to psychological distress [9].

One relatively understudied but highly relevant factor is the source through which individuals obtain conflict-related information. Different media platforms vary substantially in the frequency, emotional framing, intensity, and credibility of the information they disseminate. Social media platforms may amplify psychological distress because of continuous exposure cycles, emotionally polarized discussions, rapid dissemination of unverified information, and repeated exposure to graphic imagery. However, relatively few studies have examined whether the association between conflict-related exposure and psychological distress differs according to primary information source [8]. These issues are particularly relevant within Saudi Arabia. The Kingdom has one of the highest rates of social media utilization globally among young adults, and digital platforms represent a dominant source of news consumption among university students.

The empirical evidence examining the relationship between conflict-related news exposure and psychological distress among nursing students in Saudi Arabia remains limited. Existing studies among Saudi university students have primarily focused on conventional academic stressors, sleep quality, or the mental health effects of the COVID-19 pandemic, with limited attention given to geopolitical conflict exposure as a psychosocial determinant of mental health [10,11,12]. Furthermore, many previous investigations relied on relatively simple analytical approaches that did not account for mediating pathways, interaction effects, or the multidimensional nature of media exposure. The mental health of nursing students is influenced by a complex interaction of risk and protective factors. Recent evidence suggests that characteristics such as self-efficacy, cognitive flexibility, resilience, and adaptive coping may protect against psychological distress, whereas academic pressures, sleep disturbances, and exposure to stressful information environments may increase vulnerability [10]. Accordingly, psychological responses to conflict-related media exposure are unlikely to be explained by a single mechanism. Instead, multiple cognitive, emotional, and behavioral processes may operate simultaneously.

Several theoretical frameworks may help explain why conflict-related media exposure could influence depression, anxiety, and stress among nursing students. According to the transactional model of stress and coping, psychological responses depend not only on exposure to potentially threatening events but also on how individuals appraise their significance and personal consequences [13]. Additional pathways may involve behavioral and functional disruption. Exposure to emotionally distressing media content has been associated with heightened vigilance, intrusive thoughts, sleep disturbance, and difficulties disengaging from threat-related information, all of which may increase vulnerability to depression, anxiety, and stress [14]. The association between conflict-related media exposure and psychological distress may also be understood through theories of vicarious traumatization and emotional contagion. Among intensive care nurses, psychological inflexibility was shown to mediate the relationship between emotion regulation difficulties and perceived stress, highlighting the importance of adaptive psychological processes in determining stress responses [15]. Perceived safety concern was conceptualized as an appraisal-related indicator reflecting subjective perceptions of threat and insecurity. Unlike sleep difficulty and study disruption, which were examined as intermediary variables in exploratory analyses of indirect associations, safety concern was evaluated separately to determine whether perceptions of threat influenced the strength of the observed associations between conflict-related exposure and psychological distress.

Perceived safety concern was conceptualized as an appraisal-related construct reflecting subjective perceptions of threat and insecurity arising from exposure to conflict-related information. Within the framework of stress appraisal theory, perceived threat may influence how individuals respond emotionally to potentially distressing events.

This study was therefore designed to address these gaps using cross-sectional data collected from nursing students across multiple regions of Saudi Arabia. Specifically, this study aimed to estimate the prevalence of depression, anxiety, and stress among nursing students. We examine the association between conflict-related news exposure and psychological distress; evaluate the mediating roles of sleep difficulty and academic disruption using structural equation modeling; and assess whether the relationship between exposure and psychological outcomes differed according to primary information source.

## 2. Methods

### 2.1. Study Design and Setting

In this study, we employed a cross-sectional design. It examined the association between exposure to armed conflict-related media and psychological distress among undergraduate nursing students in Saudi Arabia. Data were collected from nursing students enrolled at different public universities located across five distinct administrative regions of Saudi Arabia. The data collection was performed from 7 to 18 April 2026. The inclusion of geographically dispersed institutions was intended to increase geographic diversity and capture nursing students from different educational and sociocultural contexts across Saudi Arabia. However, because participants were recruited using a convenience sampling approach, the sample should not be considered representative of all nursing students in Saudi Arabia, and findings should be interpreted accordingly. The five regions represented in the sample were selected to provide broad geographic coverage, thereby enhancing the external validity of findings and enabling regional comparisons in the analyses.

### 2.2. Sampling Strategy and Participants

Participants were recruited using convenience sampling. This approach is widely adopted in health and nursing education research where population-level sampling frames are unavailable or impractical to construct. Although convenience sampling introduces the possibility of selection bias, its use in exploratory and descriptive cross-sectional work among student populations is well-established and considered appropriate when the objective is to identify associations rather than make strictly generalizable population estimates. Eligibility criteria required that participants be currently enrolled as undergraduate nursing students at one of the participating universities, be aged 18 years or older, and be willing to provide voluntary informed consent. Because recruitment was conducted through institutional distribution channels and voluntary participation, the reported participation proportion should be interpreted cautiously and does not represent a response rate derived from a probability-based sampling framework.

### 2.3. Data Collection Procedure

Data were collected exclusively through an online self-administered questionnaire. Participants accessed the questionnaire through Quick Response (QR) codes. This was done using the announcement on the official notice boards in each university. The introductory page of the survey presented a plain-language information sheet explaining the study purpose, the nature of participation, the anonymity of responses, and the right to withdraw at any point without consequence. Electronic informed consent was obtained by asking participants to indicate their agreement through a confirmation checkbox before accessing the questionnaire.

### 2.4. Potential Variable Measures

Psychological distress was assessed using the Depression Anxiety Stress Scales-21 (DASS-21), a widely validated 21-item self-report instrument designed to measure the severity of core symptoms across three negative affective states: depression, anxiety, and stress [16,17]. Each of the three DASS-21 subscales comprises seven items rated on a four-point Likert scale ranging from 0 (did not apply to me at all) to 3 (applied to me very much, or most of the time), referring to the participant’s experience over the preceding week. DASS-21 subscale scores were calculated by summing responses to the seven items corresponding to each domain and multiplying the resulting scores by two, consistent with standard DASS-21 scoring procedures and to ensure comparability with the original 42-item version. Depression severity was classified as normal (0 to 9), mild (10 to 13), moderate (14 to 20), severe (21 to 27), and extremely severe (≥28). Anxiety severity was classified as normal (0 to 7), mild (8 to 9), moderate (10 to 14), severe (15 to 19), and extremely severe (≥20). Stress severity was classified as normal (0 to 14), mild (15 to 18), moderate (19 to 25), severe (26 to 33), and extremely severe (≥34). Moderate-to-severe psychological distress was defined as scores within the moderate, severe, or extremely severe categories.

The depression subscale focuses on symptoms reflecting low positive affect, anhedonia, hopelessness, and self-deprecation, including items relating to feeling downhearted, worthless, useless, and perceiving life as meaningless. The anxiety subscale captures autonomic arousal, situational anxiety, and subjective experience of anxious affect, encompassing items on breathing difficulties, palpitations, trembling, panic, and generalized worry. The stress subscale assesses difficulty relaxing, nervous arousal, and being easily upset or agitated, with items addressing restlessness, overreaction, irritability, impatience, and difficulty winding down. Internal consistency of the DASS-21 subscales was assessed using Cronbach’s alpha. Reliability was good for the depression subscale (α = 0.851) and excellent for the anxiety (α = 0.909) and stress (α = 0.917) subscales in the current sample.

Conflict-related media exposure was assessed using two questionnaire items. Participants were asked: “How closely are you following news related to ongoing armed conflicts?” with response options ranging from “Not at all” to “Extremely closely” (five-point scale), and “How frequently do you encounter conflict-related news content?” with response options ranging from “Never” to “Very often” (five-point scale). The items were selected to capture complementary dimensions of media exposure, namely engagement with conflict-related information and frequency of exposure. Both items were measured using the same response scale and were moderately correlated (r = 0.48, 95% CI: 0.38–0.57, *p* < 0.001). Responses were summed to create a composite exposure score and subsequently standardized using z-score transformation, resulting in a continuous exposure index with a mean of zero and standard deviation of one. Standardization was performed to facilitate interpretation of regression coefficients and comparability across analyses.

Perceived safety concern was assessed using a single item asking participants about their level of concern regarding their own or their family’s safety in relation to ongoing conflicts, with responses ranging from “Not concerned” to “Extremely concerned”. Sleep difficulty was assessed using a single item measuring the frequency of sleep difficulties attributed to conflict-related news exposure, while study disruption was measured using a single item assessing the extent to which conflict-related news affected the participant’s ability to concentrate on academic activities. Both variables were rated on five-point ordinal scales. These measures were included as pragmatic indicators of cognitive and behavioral responses to conflict-related media exposure and should not be interpreted as validated multidimensional constructs.

We also adjusted for several covariates, including gender (male/female), age (categorized as 18–20, 21–23, 24–26, 27–30, and above 30 years), marital status (single, married, divorced, widowed), living arrangement (with family, alone, with roommate, university housing), academic year of study (first through fourth year and internship), and self-reported cumulative grade point average (GPA) categorized on the Saudi five-point scale. Geographic region was captured through the participant’s affiliated university, which was coded into five regional categories corresponding to the institutional sites. The primary information source for conflict-related news was assessed with a single item asking participants to indicate their main channel for obtaining news about ongoing armed conflicts, with response categories including television news, social media platforms, online news websites, friends and family, and not following news.

### 2.5. Statistical Analysis

Descriptive statistics were conducted for all sociodemographic variables and mental health outcomes, stratified by gender. Continuous variables were summarized as means and standard deviations, and categorical variables as frequencies and percentages. Prevalence of depression, anxiety, and stress was estimated as the proportion of participants meeting or exceeding the respective DASS-21 thresholds described above. A Pearson correlation matrix was computed to characterize bivariate relationships among continuous variables.

#### 2.5.1. Gamma Regression Models

Given the right-skewed distribution of DASS-21 subscale scores and the potential for the linear regression assumptions of normality and constant variance to be violated in this context, we performed Gamma generalized linear models (GLMs) with a log link function [18]. Gamma regression requires strictly positive outcome values; therefore, we added a constant value of one to each DASS-21 subscale score prior to analysis to accommodate participants with scores of zero while retaining all observations in the dataset. Two nested Gamma models were estimated for each outcome: Two nested models were estimated for each outcome. Model 1 (the base model) included the conflict exposure index, primary information source, gender, age category, GPA category, and university region as predictors. Model 2 extended Model 1 by additionally adjusting for safety concern score, which was included as a candidate mediating or confounding variable to assess the degree to which the exposure–outcome relationship was attenuated after controlling for this pathway. Exponentiated coefficients (arithmetic mean ratios, AMRs) with 95% confidence intervals are reported.

#### 2.5.2. Structural Equation Modelling

To examine potential indirect pathways through which conflict exposure may affect psychological outcomes, we performed structural equation models (SEMs). Structural equation models were estimated using the lavaan package in R (version 4.4.3). Separate structural equation models were fitted for depression, anxiety, and stress. In each model, conflict-related exposure was specified as the predictor, while sleep difficulty and study disruption were included as intermediary variables. Standardized path coefficients were estimated for direct and indirect associations. Indirect associations were evaluated using standardized path coefficients and their associated *p*-values; bootstrapped confidence intervals were not computed. The SEM analyses were exploratory and intended to evaluate whether the observed data were consistent with hypothesized indirect relationships. Given the cross-sectional nature of the study, the analyses should not be interpreted as establishing temporal ordering or causal mediation. Standardized path coefficients (β) and associated *p*-values are reported for each pathway across all three outcomes.

#### 2.5.3. Interaction Analysis

We also further examine whether the association between conflict exposure and psychological distress varied according to participants’ primary information source, multiplicative interaction terms between the exposure index and information source category were introduced into the Gamma regression models. Separate interaction models were fitted for depression, anxiety, and stress. To account for multiple comparisons across the three outcomes, Bonferroni correction was applied to the resulting *p*-values.

## 3. Results

A total of 247 nursing students participated in the study, corresponding to a response rate of 18%, with a balanced distribution of males (50.2%) and females (49.8%) (Table 1). Nearly half of the sample were aged 21–23 years (49.5%), followed by those aged 24–26 years (21.5%). Most participants lived with their families (64.0%), whereas 15.0% lived alone, 9.1% with roommates, and 12.5% in university housing. Most respondents are in their third year. Over half of the students reported a GPA of 4.0 or above (54.5%), with 20.0% achieving a GPA between 4.5 and 5.0. Only 3.7% reported a GPA below 2.0. Respondents answer that media was the most commonly reported source of conflict-related news (36.0%), followed by television (22.5%) and online news platforms (21.0%). Approximately 11.0% reported not following conflict-related news. The average depression score was 13.5, anxiety was 14.0, and stress was 12.5. Female students showed slightly higher mean scores for depression and stress compared to males.

We also determined the prevalence, and found values of 50.2%, 59.9% and 32.4% for depression, anxiety and stress, respectively.

Correlation analysis showed strong positive associations among depression, anxiety, and stress (r = 0.91–0.93), indicating substantial overlap between psychological distress domains (Table S1). Exposure to conflict-related news was modestly correlated with distress outcomes (r = 0.24–0.270. In contrast, sleep difficulty and study disruption exhibited stronger correlations with depression, anxiety, and stress (r = 0.53–0.57). Additionally, exposure was moderately associated with both sleep difficulty and study disruption (r = 0.29). This supports a potential indirect pathway linking exposure to psychological distress through behavioral disruption.

**Figure 1:**
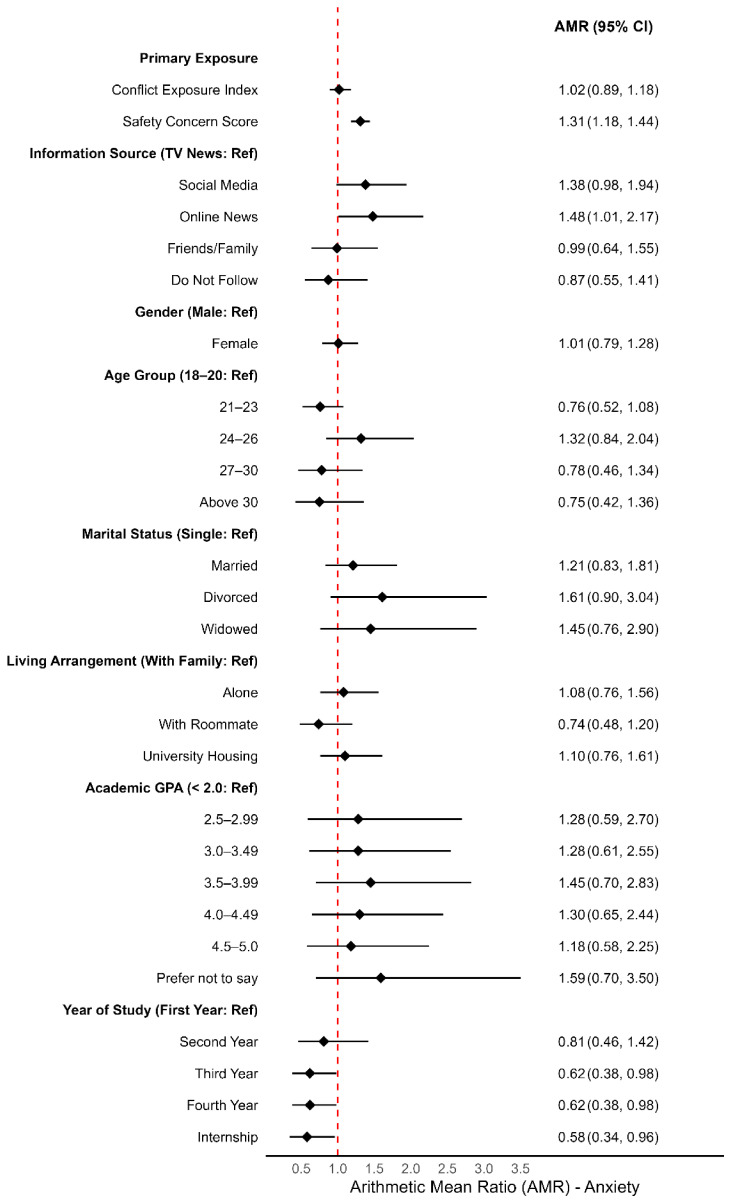

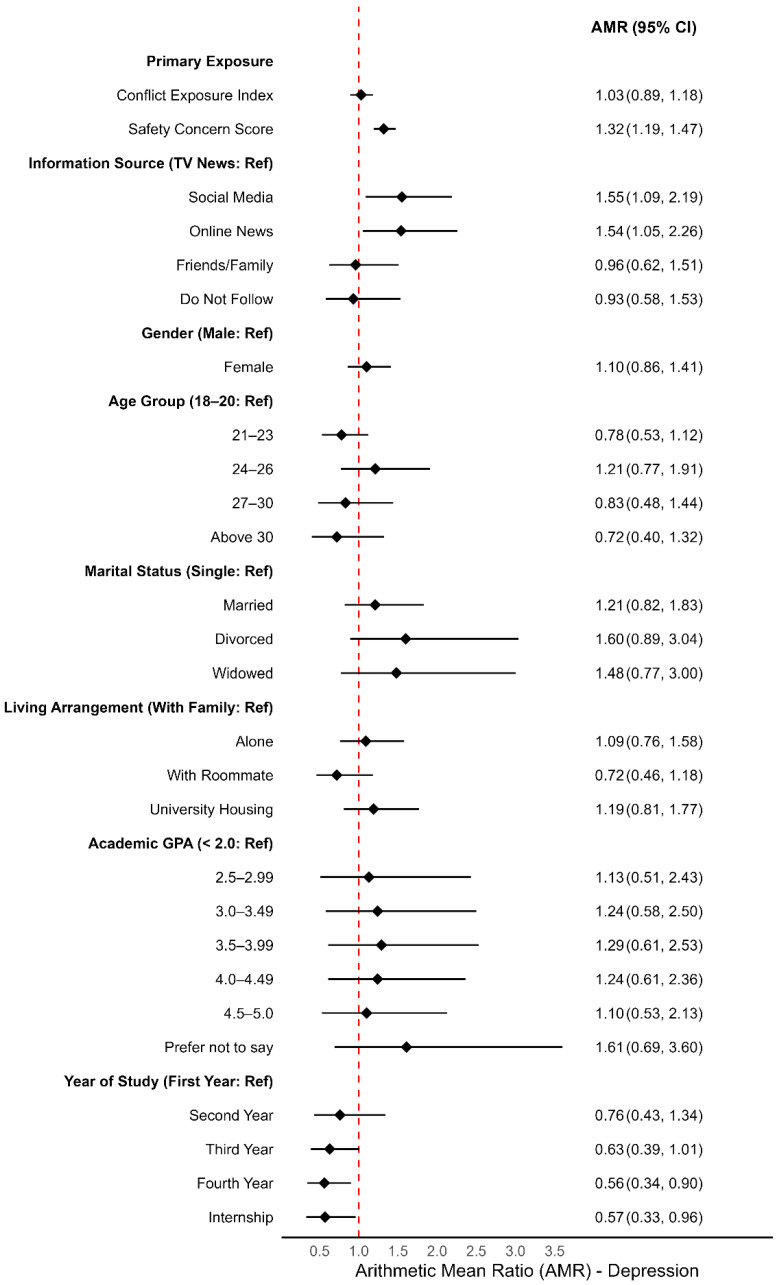

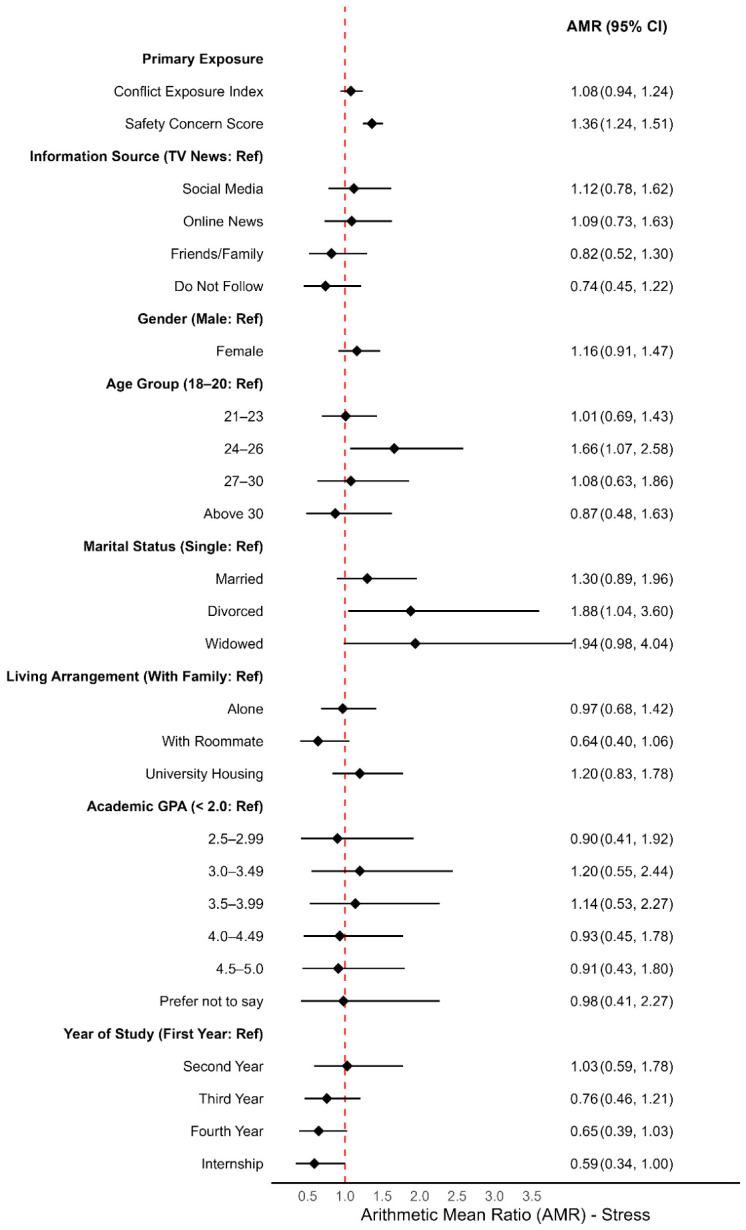
Forest plot of adjusted Arithmetic Mean Ratios (AMR) for predictors of (Anxiety: Top panel; Depression: middle panel; Stress: bottom panel). The diamond markers represent the AMR, and the horizontal error bars represent the 95% Confidence Intervals (CIs). The vertical dashed red line denotes the AMR = 1.0; estimates to the right of the line indicate a positive association, while those to the left indicate a negative association. Categorical variables include the reference group in parentheses.

Figure 1 presents the adjusted associations between conflict-related news exposure and psychological distress across anxiety (top panel), depression (middle panel) and stress (bottom panel). Our study, after accounting for perceived safety concerns and other covariates, the exposure index was not significantly associated with depression (AMR 1.03, 95% CI 0.89 to 1.18), anxiety (AMR 1.02, 95% CI 0.89 to 1.18), or stress (AMR 1.08, 95% CI 0.94 to 1.24). We found that there was a strong association between safety concern and health outcomes. Each unit increase in safety concern was associated with higher levels of depression (AMR 1.32, 95% CI 1.19 to 1.47), anxiety (AMR 1.31, 95% CI 1.18 to 1.44), and stress (AMR 1.36, 95% CI 1.24 to 1.51). There was a consistent magnitude of estimates observed across outcomes, indicating a strong and stable association.

Information source also showed strong association. Compared with TV news, social media use was associated with higher depression (AMR 1.55, 95% CI 1.09 to 2.19) and showed a borderline association with anxiety (AMR 1.38, 95% CI 0.98 to 1.94). Online news was associated with higher depression (AMR 1.54, 95% CI 1.05 to 2.26) and higher anxiety (AMR 1.48, 95% CI 1.01 to 2.17). No strong association was found for stress. Information from friends or family and not following news were not associated with any outcome.

The results without adjustment for safety concern provides important implication (Supplementary Table S2). Our findings showed that exposure was associated with higher distress, particularly for stress (AMR 1.17, 95% CI 1.02 to 1.34), with similar but less precise estimates for depression (AMR 1.14, 95% CI 0.99 to 1.30) and anxiety (AMR 1.13, 95% CI 0.99 to 1.28). After inclusion of safety concern in the main models, these associations attenuated. This pattern indicates that perceived safety concern explains a substantial portion of the relationship between exposure and psychological distress.

The role of information source remained evident across both analyses. In the supplementary models, the effects were stronger, particularly for online news, which was associated with nearly twofold higher depression (AMR 1.92, 95% CI 1.32 to 2.78), anxiety (AMR 1.84, 95% CI 1.29 to 2.64), and stress (AMR 1.88, 95% CI 1.29 to 2.74). These estimates decreased but remained elevated in the fully adjusted models, suggesting both direct effects and indirect effects through safety concern.

There were consistent findings for other sociodemographic factors. Gender was not associated with any outcome. Age was associated with stress only, with higher stress among those aged 24–26 (AMR 1.66, 95% CI 1.07 to 2.58). Marital status showed an association with stress, with higher levels among divorced (AMR 1.88, 95% CI 1.04 to 3.60). Living in university housing was associated with higher depression and stress. Academic GPA was not associated with psychological distress. The year of study showed a consistent protective pattern, with lower depression and anxiety among students in later years, particularly third, fourth, and internship levels.

We have also checked for multicollinearity. All adjusted GVIF values were close to 1, indicating very weak correlations among predictors (Table S5).

There was significant effect modification by information source in the association between conflict-related news exposure and psychological distress (Supplementary Table S4). We observed consistently. Evidence of effect modification by information source was observed, particularly for depression. The interaction coefficients indicated that the exposure-distress association differed across information sources relative to television news; however, interpretation of these coefficients should consider the accompanying main effects. Predicted values demonstrated that students relying on social media generally reported higher levels of psychological distress across the exposure range, whereas differences between information sources varied according to outcome and exposure level. These findings suggest that the relationship between conflict-related exposure and psychological distress may differ across media environments, although the magnitude and direction of these differences should be interpreted cautiously given the relatively small sample size within some information-source categories.

There was a comparable pattern for anxiety and stress, with reduced effect sizes across these sources.

Likelihood ratio tests supported the presence of interaction for depression (*p* = 0.011), which remained statistically significant after Bonferroni correction (adjusted *p* = 0.034) (Supplementary Table S3). Evidence for interaction was weaker for stress (*p* = 0.017; adjusted *p* = 0.052) and not statistically significant for anxiety after correction (*p* = 0.043; adjusted *p* = 0.129). Our findings showed that there was no significant modification observed among students who reported not following conflict-related news.

**Figure 2:**
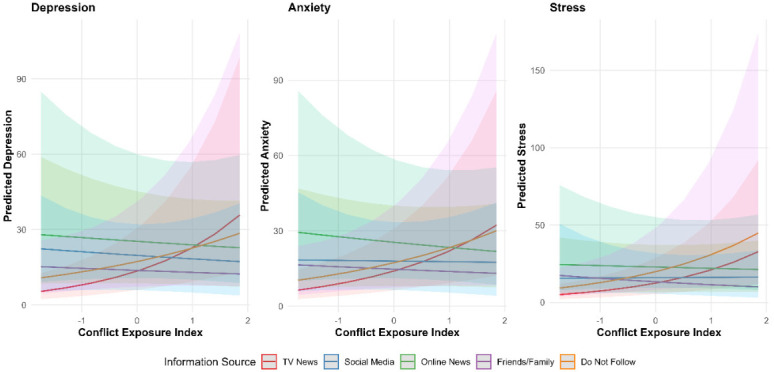
Interaction between conflict-related news exposure and information source across psychological distress outcomes. Predicted values for depression, anxiety, and stress. Lines represent adjusted mean estimates, and shaded areas indicate 95% confidence intervals.

The interaction coefficients represent differences in the exposure-distress association relative to television news, whereas the predicted values shown in Figure 2 reflect the combined influence of both the main effects and interaction effects. Accordingly, differences in predicted outcome levels may reflect variation in baseline distress, exposure-response slopes, or both. Predicted value also showed that higher conflict exposure was associated with increased psychological distress, but the magnitude of this association varied by information source (Figure 2). The steepest gradients were observed for social media, where depression, anxiety, and stress scores increased with higher exposure levels. In contrast, TV news and online news showed flatter or attenuated trends, while friends and family sources remained relatively stable across exposure levels. Confidence intervals were wider for some information-source categories, particularly those with fewer participants, indicating greater uncertainty around the corresponding predicted estimates.

We found a strong association of exposure to conflict-related news and both mediators (sleep difficulty and study disruption) (Table 2). A one standard deviation increase in exposure corresponded to higher sleep difficulty (β = 0.29, *p* < 0.001) and greater study disruption (β = 0.29, *p* < 0.001). These effects were identical across depression, anxiety, and stress, indicating a stable upstream pathway. For the direct effects of sleep difficulty, we observed strong associations across all outcomes with standardized coefficients of 0.38 for depression, 0.39 for anxiety, and 0.41 for stress (all *p* < 0.001). Study disruption also demonstrated consistent associations, with effects ranging from 0.32 to 0.33 (all *p* < 0.001). This indicates that sleep disturbance is the dominant pathway, while academic disruption provides an additional, independent contribution.

The direct effect of exposure on outcomes was small and not statistically significant across all models (β = 0.05 to 0.08, *p* = 0.39). In contrast, the indirect effects were substantial and highly significant, with standardized estimates of 0.20 for depression and 0.21 for both anxiety and stress (all *p* < 0.001). Therefore, our findings suggested that the observed associations between exposure and psychological distress were largely consistent with indirect associations involving sleep difficulty and study disruption, whereas direct associations were comparatively weak.

The model was just-identified or nearly just-identified. As a result, global fit indices are not informative and were not interpreted. In this context, inference relies on the magnitude and consistency of the path coefficients, which demonstrate a coherent and theoretically grounded mediation structure.

## 4. Discussion

In this study, we examined the psychological impact of conflict related news exposure among nursing students in Saudi Arabia during the February 2026 regional military escalation involving Iran. The findings demonstrated a substantial burden of psychological distress within the study population, with approximately half of participants meeting criteria for moderate to severe depression, nearly 60% reporting moderate to severe anxiety, and one third reporting moderate to severe stress symptoms. Conflict related exposure was associated with worse mental health outcomes, particularly stress, while perceived safety concern emerged as a major explanatory factor underlying these relationships. Sleep difficulty and study disruption further mediated the association between exposure and psychological distress, whereas the relationship between exposure and distress differed according to primary information source. Our findings suggest that conflict-related media exposure represents an important and multidimensional psychosocial stressor among nursing students in Saudi Arabia.

In our study, the prevalence of psychological distress observed in this study was notably high. We found that approximately 50.2% of students reported moderate to severe depressive symptoms. A recent study among nursing students similarly reported high depressive symptom prevalence and identified psychological vulnerability associated with problematic social media use [19]. A review on the association of social media use and psychological distress also emphasized that nursing students who use social media a lot are more likely to report psychological distress [20]. Several potential mechanisms may help explain why social media users exhibited higher predicted levels of psychological distress, including algorithmic amplification of emotionally salient content, more frequent exposure to graphic imagery, rapid information-sharing cycles, and increased exposure to misinformation. However, these factors were not directly assessed in the present study and therefore should be considered speculative explanations that warrant further investigation.

One of the main findings of this study was the strong association between conflict-related news exposure and psychological distress among nursing students. However, this association became substantially attenuated after accounting for perceived safety concern, while safety concern itself remained strongly associated with depression, anxiety, and stress. This pattern suggests that the psychological effects of conflict-related media exposure may operate primarily through increased perceptions of insecurity and threat rather than conflict-related news alone. Previous studies demonstrated that media-mediated exposure to armed conflict was associated with increased anxiety, stress, and depressive symptoms, particularly among individuals experiencing elevated perceptions of insecurity and uncertainty [8,21,22]. Our findings may be especially relevant among nursing students because healthcare training promotes high empathic sensitivity and emotional engagement with human suffering, potentially increasing vulnerability to stress responses during periods of regional geopolitical instability [23].

In this study, the findings regarding information source were particularly important. Compared with students relying primarily on television news, those using social media and online news platforms consistently demonstrated higher levels of psychological distress. The interaction results further demonstrated steeper increases in predicted psychological distress among students primarily using social media and online news sources compared with traditional television news users. A systematic review based on 21 cross-sectional studies from Arab countries suggested that there were strong association between problematic social media use and mental health [24]. It also further emphasized that academic interference and sleep disruption mediate the association. The association was also reported in other studies [25,26]. A study among medical students in Iran additionally demonstrated that excessive mobile social network use was significantly associated with poorer mental health and maladaptive social behaviors [27]. Several mechanisms may explain why social media and online news exposure appeared more psychologically harmful than television news exposure in our study. Social media platforms provide continuous and repetitive exposure to emotionally intense content, frequently accompanied by graphic imagery, sensational framing, emotionally polarized discussions, and unverified information. Unlike television broadcasts, which are generally structured and moderated through editorial processes, social media platforms deliver rapid and uninterrupted information cycles that may sustain emotional arousal and uncertainty. Algorithm-driven amplification further increases exposure frequency to emotionally engaging content, reinforcing fear-related cognitions and stress responses [28]. Studies examining digital media environments have similarly suggested that repeated exposure to emotionally negative online content contributes to emotional dysregulation, heightened anxiety, and chronic stress reactions [29].

The structural equation modeling findings suggest that the psychological effects of conflict-related media exposure may operate primarily through sleep difficulty and academic interference, rather than through direct exposure alone. This interpretation is strongly supported by previous literature demonstrating that sleep disturbance represents one of the most important predictors of depression, anxiety, and stress among university students and healthcare trainees [24]. Exposure to emotionally distressing and threatening information may impair sleep initiation, increase nocturnal rumination, and reduce emotional recovery capacity, thereby increasing vulnerability to psychological distress [30]. Previous studies among nursing students have consistently identified academic burden, emotional exhaustion, and impaired study functioning as major determinants of poor mental health outcomes [20,31]. The findings additionally support theories of vicarious traumatization and emotional contagion, which propose that repeated exposure to conflict-related suffering through media may generate secondary traumatic stress responses even among indirectly exposed individuals [32]. However, the indirect effects identified in the SEM analyses should be interpreted cautiously. Because exposure, intermediary variables, and psychological outcomes were measured simultaneously, the findings do not establish temporal sequencing or causal mediation. Rather, they indicate that the observed associations were statistically consistent with indirect relationships involving sleep difficulty and study disruption.

Interestingly, several sociodemographic variables demonstrated relatively limited associations with psychological outcomes after adjustment for exposure-related factors. Students residing in university housing, however, consistently demonstrated increased psychological distress. This finding may reflect reduced family support, increased social isolation, or greater exposure to digital media environments within residential university settings. Previous studies have similarly shown that social support and living conditions substantially influence psychological resilience among nursing students and healthcare workers [33].

Our findings suggest that the psychological impact of conflict-related media exposure extends beyond simple information consumption and may reflect broader psychological processes related to threat perception and coping. The attenuation of the exposure estimates after adjustment for safety concern suggests that perceived threat and insecurity may be relevant to understanding the association between conflict-related exposure and psychological distress. However, because safety concern was not formally evaluated within a mediation framework and all variables were measured concurrently, these findings should be interpreted cautiously. The observed attenuation may reflect appraisal-related processes, shared variance among self-reported measures, or other unmeasured factors rather than a specific mediating mechanism. This interpretation is consistent with evidence showing that perceived threat is a major determinant of psychological distress during periods of geopolitical instability [14]. Furthermore, the observed mediation through sleep difficulty and academic disruption suggests that exposure may influence mental health indirectly by interfering with daily functioning and emotional recovery. Similar findings have been reported among healthcare professionals, where difficulties in emotion regulation and reduced psychological flexibility were associated with greater stress and poorer psychological well-being under prolonged stress exposure [15]. Our findings support the view that repeated exposure to conflict-related content may contribute to psychological distress through interconnected cognitive and behavioral pathways rather than through direct exposure alone.

### Strength and Limitations

This study has several important strengths. The investigation was conducted across several universities located in five regions of Saudi Arabia, which improved geographic diversity and enhanced representation of nursing students from different educational contexts within the country. The study was performed during an active period of regional geopolitical escalation involving Iran, allowing assessment of psychological responses in a real-time conflict-related information environment rather than through retrospective recall of distant events. The analysis approach was comprehensive and incorporated multiple complementary statistical techniques, including multivariable Gamma regression, interaction analysis, mediation analysis, and structural equation modeling, which enabled evaluation of both direct and indirect pathways linking conflict-related exposure to psychological distress. We further examined moderation by information source, an area that remains insufficiently explored in the literature despite the growing importance of digital media exposure in shaping psychological wellbeing. The inclusion of sleep difficulty, study disruption, and perceived safety concern further allowed exploration of plausible behavioral and cognitive mechanisms underlying the observed associations.

Several limitations should be acknowledged. We cannot interpret the causal effects due to the cross-sectional study design. The use of convenience sampling and the relatively low response rate may have introduced substantial self-selection bias. Students experiencing greater psychological distress, heightened concern regarding regional conflicts, or stronger engagement with conflict-related media may have been more likely to participate, potentially influencing prevalence estimates and observed associations. Because information on non-respondents was unavailable, we were unable to evaluate the extent of non-response bias or compare respondents with the underlying source population. Although participants were recruited from universities located across multiple regions of Saudi Arabia, the sample was not probability-based and should not be considered nationally representative. Consequently, the findings should be interpreted as exploratory and may not be fully generalizable to all nursing students in Saudi Arabia or other settings. The use of self-reported measures collected within a single survey may have introduced common-method bias, potentially inflating correlations among exposure, intermediary variables, and psychological outcomes. Reverse causation is also possible, as students experiencing greater psychological distress may have been more likely to follow conflict-related news, report heightened safety concerns, or perceive greater disruption to sleep and academic functioning. Sleep difficulty and study disruption were assessed using single-item measures, which may be less reliable and less comprehensive than validated multi-item instruments. The conflict exposure index was derived from two self-reported items designed to capture engagement with and frequency of conflict-related media exposure, and has not undergone formal psychometric validation. Furthermore, safety concern, sleep difficulty, and study disruption were assessed using single-item indicators. Although these measures were selected to reduce respondent burden and capture key constructs relevant to the study objectives, they may be more susceptible to measurement error than validated multi-item scales. The study additionally did not assess pre-existing psychiatric disorders, prior trauma exposure, personality characteristics, coping strategies, resilience factors, social support, religiosity, financial stress, academic workload, or concurrent life stressors, all of which could influence psychological outcomes and potentially confound observed associations. Future studies should incorporate these variables to provide a more comprehensive understanding of the psychological, social, and contextual factors shaping mental health responses to conflict-related media exposure. Although the structural equation models provided insight into potential mediation pathways, the models were relatively simple and primarily exploratory in nature. Because the models were nearly just identified, global fit indices should be interpreted cautiously. Participants were asked to report the extent to which these experiences were related to conflict-related news exposure. Consequently, students experiencing higher levels of psychological distress may have been more likely to attribute sleep difficulties or academic challenges to conflict-related content, potentially contributing to common-method and attribution biases. The timing of data collection may also have influenced the findings. Psychological responses during periods of active geopolitical escalation are likely dynamic and may fluctuate according to media intensity, political developments, and regional security perceptions. Although depression, anxiety and stress were analyzed separately in accordance with the DASS framework and study objectives, the high intercorrelations suggest that the observed associations may partly reflect a broader underlying dimension of psychological distress. Consequently, the consistency of findings across all three outcomes may indicate that conflict-related exposure is associated with general psychological distress rather than exclusively with any single domain. Finally, because the study was conducted within Saudi Arabia, cultural, political, and regional contextual factors may influence the generalizability of the findings to populations outside the Middle East.

## 5. Conclusions

This study found a high prevalence of depression, anxiety, and stress among nursing students during a period of regional geopolitical tension. Although greater conflict-related news exposure was associated with psychological distress in preliminary analyses, these associations were no longer statistically significant after accounting for perceived safety concern and other covariates. In contrast, perceived safety concern remained consistently associated with depression, anxiety, and stress.

Higher exposure was also associated with greater safety concern, sleep difficulty, and academic disruption, which were statistically linked to psychological distress in exploratory mediation analyses. Although the cross-sectional design precludes conclusions regarding causality, the findings highlight the potential importance of psychological wellbeing, sleep health, and media-related experiences during periods of regional geopolitical instability. Universities may consider strengthening student support services, mental health resources, and media literacy initiatives, while future longitudinal studies are needed to determine whether these factors contribute to changes in psychological distress over time.

## Figures and Tables

**Table 1:** Distribution of sociodemographic characteristics, conflict-related information sources, and mean psychological distress scores among university students.

Characteristics	Male (n = 124)	Female (n = 123)	Total (n = 247)
Age Group, n (%)			
18–20	10 (8.1%)	23 (19.0%)	33 (13.4%)
21–23	67 (54.0%)	55 (45.0%)	122 (49.4%)
24–26	28 (23.0%)	25 (20.0%)	53 (21.5%)
27–30	12 (9.7%)	10 (8.1%)	22 (8.9%)
Above 30	7 (5.6%)	10 (8.1%)	17 (6.9%)
Living Arrangement, n (%)			
With Family	80 (65.0%)	77 (63.0%)	157 (63.6%)
Alone	18 (15.0%)	19 (15.0%)	37 (15.0%)
With Roommate	9 (7.3%)	13 (11.0%)	22 (8.9%)
University Housing	17 (14.0%)	14 (11.0%)	31 (12.6%)
Year of Study, n (%)			
First Year	11 (8.9%)	11 (8.9%)	22 (8.9%)
Second Year	10 (8.1%)	20 (16.0%)	30 (12.1%)
Third Year	55 (44.0%)	36 (29.0%)	91 (36.8%)
Fourth Year	31 (25.0%)	35 (28.0%)	66 (26.7%)
Internship	17 (14.0%)	21 (17.0%)	38 (15.4%)
Academic GPA, n (%)			
<2.0	5 (4.0%)	4 (3.3%)	9 (3.6%)
2.5–2.99	6 (4.8%)	11 (8.9%)	17 (6.9%)
3.0–3.49	22 (18.0%)	13 (11.0%)	35 (14.2%)
3.5–3.99	27 (22.0%)	10 (8.1%)	37 (15.0%)
4.0–4.49	42 (34.0%)	43 (35.0%)	85 (34.4%)
4.5–5.0	18 (15.0%)	31 (25.0%)	49 (19.8%)
Prefer not to say	4 (3.2%)	11 (8.9%)	15 (6.1%)
Information Source, n (%)			
TV News	21 (17.0%)	30 (24.0%)	51 (20.6%)
Social Media	45 (36.0%)	44 (36.0%)	89 (36.0%)
Online News	31 (25.0%)	21 (17.0%)	52 (21.1%)
Friends/Family	13 (10.0%)	15 (12.0%)	28 (11.3%)
Do Not Follow	14 (11.0%)	13 (11.0%)	27 (10.9%)
Psychological Outcomes, Mean (SD)			
Depression	13.0 (11.0)	14.0 (11.0)	13.5 (11.0)
Anxiety	14.0 (11.0)	14.0 (11.0)	14.0 (11.0)
Stress	12.0 (11.0)	13.0 (11.0)	12.5 (11.0)

**Table 2:** Structural equation modeling of the association between conflict-related exposure and psychological distress. Values are standardized coefficients (β) with *p*-values. Indirect effects represent the combined mediation pathways through sleep difficulty and study disruption.

Pathway	Depression	Anxiety	Stress
Exposure → Sleep difficulty	0.29 (<0.001)	0.29 (<0.001)	0.29 (<0.001)
Exposure → Study disruption	0.29 (<0.001)	0.29 (<0.001)	0.29 (<0.001)
Sleep difficulty → Outcome	0.38 (<0.001)	0.39 (<0.001)	0.41 (<0.001)
Study disruption → Outcome	0.33 (<0.001)	0.32 (<0.001)	0.32 (<0.001)
Direct effect (Exposure → Outcome)	0.05 (0.391)	0.08 (0.183)	0.05 (0.390)
Indirect effect (via sleep and study)	0.20 (<0.001)	0.21 (<0.001)	0.21 (<0.001)

## Data Availability

The data presented in this study are available on request from the corresponding author. The data are not publicly available due to privacy or ethical restrictions.

## Supplementary Materials

The following supporting information can be downloaded at: https://www.mdpi.com/article/10.3390/healthcare14121609/s1, Table S1: presents Pearson correlation coefficients among psychological distress outcomes, exposure to conflict-related news, and behavioral disruption variables. Table S2: shows associations between conflict-related news exposure and psychological distress without adjustment for perceived safety concern using Gamma regression models. Tables S3 and S4: present interaction analyses between conflict exposure and information source across outcomes using Gamma regression models, with Table S4 reporting adjusted mean ratios (AMRs). Table S5: provides an assessment of multicollinearity across Gamma regression models using adjusted variance inflation factors (GVIF_adj).
