# Supplementary material for: Exposure to Conflict-Related News and Psychological Distress Among Nursing Students: The Mediating Role of Sleep Difficulties and Study Disruption
Source: Healthcare (Basel). 2026 Jun 8;14(12):1609. doi: 10.3390/healthcare14121609 (PMC13299654; doi:10.3390/healthcare14121609)
Supplement: Supplementary file 1 [file healthcare-14-01609-s001.zip › healthcare-4345694-supplementary.pdf]

Supplementary results

Exposure to Conflict-Related News and Psychological Distress Among Nursing Students: The Mediating Role of Sleep Difficulties and Study Disruption

Majed M. Aljabri [1,*], Bandar S. Alharbi [1] and Endale Alemayehu Ali [2,*]

[1] Community and Psychiatric Mental Health Department, College of Nursing, King Saud University,
Riyadh 12375, Saudi Arabia; banalharbi@ksu.edu.sa
[2] Department of Public Health and Primary Care, KU Leuven, Kapucijnenvoer 33, 3000 Leuven, Belgium

* Correspondence: amajad@ksu.edu.sa (M.M.A.); endalestat@gmail.com (E.A.A.)

Table S1. Pearson correlation coefficients among psychological distress outcomes, exposure to conflict-related news, and behavioral disruption variables.

Variable	1	2	3	4	5	6
1. Depression	—					
2. Anxiety	0.93	—				
3. Stress	0.91	0.91	—			
4. Conflict Exposure Index	0.24	0.27	.24	—		
5. Sleep Difficulties	0.55	0.57	0.57	0.29	—	
6. Academic Impact	0.53	0.54	0.53	0.29	0.58	—

Table S2. Associations between conflict-related news exposure and psychological distress without adjustment for perceived safety concern (Gamma regression models).

Predictors	Depression AMR (95% CI)	Anxiety AMR (95% CI)	Stress AMR (95% CI)
Primary Exposure			
Conflict Exposure Index	1.14 (0.99, 1.30)	1.13 (0.99, 1.28)	1.17 (1.02, 1.34)
Information Source			
TV News	—	—	—
Social Media	1.56 (1.11, 2.19)	1.37 (0.99, 1.89)	1.38 (0.99, 1.93)
Online News	1.92 (1.32, 2.78)	1.84 (1.29, 2.64)	1.88 (1.29, 2.74)
Friends/Family	1.14 (0.75, 1.77)	1.17 (0.77, 1.78)	1.22 (0.79, 1.91)
Do Not Follow	1.31 (0.81, 2.12)	1.20 (0.77, 1.90)	1.40 (0.87, 2.29)
Gender			
Male	—	—	—
Female	1.10 (0.86, 1.41)	1.01 (0.80, 1.28)	1.15 (0.90, 1.47)
Age Group			
18–20	—	—	—
21–23	0.79 (0.53, 1.14)	0.78 (0.53, 1.10)	0.93 (0.63, 1.35)
24–26	1.20 (0.76, 1.89)	1.32 (0.85, 2.03)	1.47 (0.93, 2.32)
27–30	0.73 (0.42, 1.28)	0.72 (0.42, 1.24)	0.94 (0.54, 1.66)
Above 30	0.61 (0.35, 1.11)	0.66 (0.38, 1.19)	0.73 (0.40, 1.35)
Marital Status			

Single	—	—	—
Married	1.20 (0.80, 1.83)	1.18 (0.80, 1.75)	1.15 (0.78, 1.75)
Divorced	1.30 (0.73, 2.45)	1.21 (0.70, 2.20)	1.41 (0.77, 2.73)
Widowed	1.15 (0.60, 2.31)	1.10 (0.59, 2.16)	1.16 (0.60, 2.39)
Living Arrangement			
With Family	—	—	—
Alone	1.18 (0.82, 1.70)	1.18 (0.84, 1.68)	1.15 (0.80, 1.68)
With Roommate	0.80 (0.50, 1.31)	0.87 (0.56, 1.38)	0.82 (0.51, 1.34)
University Housing	1.52 (1.05, 2.25)	1.41 (0.99, 2.03)	1.60 (1.11, 2.38)
Academic GPA			
< 2.0	—	—	—
2.5–2.99	1.09 (0.49, 2.33)	1.24 (0.57, 2.58)	0.84 (0.37, 1.83)
3.0–3.49	1.09 (0.52, 2.18)	1.08 (0.52, 2.09)	1.01 (0.47, 2.07)
3.5–3.99	1.42 (0.68, 2.78)	1.50 (0.74, 2.88)	1.15 (0.54, 2.30)
4.0–4.49	1.27 (0.62, 2.40)	1.24 (0.63, 2.28)	0.88 (0.42, 1.70)
4.5–5.0	1.19 (0.58, 2.31)	1.21 (0.60, 2.28)	0.95 (0.45, 1.89)
Prefer not to say	2.04 (0.87, 4.64)	1.78 (0.79, 3.93)	1.31 (0.53, 3.11)
Year of Study			
First Year	—	—	—
Second Year	0.95 (0.54, 1.65)	1.03 (0.61, 1.73)	1.12 (0.64, 1.94)
Third Year	0.70 (0.43, 1.10)	0.68 (0.43, 1.05)	0.74 (0.45, 1.19)
Fourth Year	0.60 (0.36, 0.96)	0.65 (0.41, 1.01)	0.65 (0.40, 1.05)
Internship	0.74 (0.44, 1.24)	0.74 (0.45, 1.20)	0.79 (0.46, 1.32)

Table S3. Interaction Between Conflict Exposure and Information Source Across Outcomes (Gamma Regression).

Outcome	Model Comparison	Δ Deviance	df	p-value	Bonferroni-adjusted p	Interpretation
Depression	Base vs Interaction	10.01	4	0.011	0.034	Significant interaction
Anxiety	Base vs Interaction	7.08	4	0.043	0.129	Not significant after correction
Stress	Base vs Interaction	9.59	4	0.017	0.052	Borderline

Table S4. Interaction Between Conflict Exposure and Information Source Across Outcomes (Gamma Models, AMR).

Interaction Term	Depression AMR (95% CI)	Anxiety AMR (95% CI)	Stress AMR (95% CI)
Exposure × Social media	0.55 (0.39–0.76)	0.63 (0.45–0.87)	0.60 (0.43–0.85)
Exposure × Online news	0.56 (0.37–0.83)	0.59 (0.40–0.87)	0.57 (0.38–0.88)
Exposure × Friends/family	0.56 (0.34–0.91)	0.60 (0.37–0.97)	0.51 (0.31–0.82)
Exposure × Do not follow	0.77 (0.48–1.24)	0.86 (0.55–1.34)	0.92 (0.57–1.48)

Table S5. Assessment of multicollinearity using adjusted variance inflation factors (GVIF_adj) across Gamma regression models.

Predictor	Depression (GVIF_adj)	Anxiety (GVIF_adj)	Stress (GVIF_adj)
Exposure index	1.24	1.24	1.24
Safety concern	1.19	1.19	1.19
Information source	1.12	1.12	1.12
Gender	1.11	1.11	1.11
Age	1.15	1.15	1.15
Marital status	1.15	1.15	1.15
Living arrangement	1.12	1.12	1.12

GPA	1.08	1.08	1.08
Year of study	1.10	1.10	1.10

Notes: Values represent adjusted generalized variance inflation factors (GVIF_adj). All values are well below commonly used thresholds for multicollinearity (GVIF_adj < 2), indicating no evidence of problematic collinearity among predictors across models.
